# Profiling Cellular Protein Complexes by Proximity Ligation with Dual Tag Microarray Readout

**DOI:** 10.1371/journal.pone.0040405

**Published:** 2012-07-10

**Authors:** Maria Hammond, Rachel Yuan Nong, Olle Ericsson, Katerina Pardali, Ulf Landegren

**Affiliations:** Department of Immunology, Genetics and Pathology, Science for Life Laboratory, Rudbeck Laboratory, Uppsala University, Uppsala, Sweden; Université Paris-Diderot, France

## Abstract

Patterns of protein interactions provide important insights in basic biology, and their analysis plays an increasing role in drug development and diagnostics of disease. We have established a scalable technique to compare two biological samples for the levels of all pairwise interactions among a set of targeted protein molecules. The technique is a combination of the proximity ligation assay with readout via dual tag microarrays. In the proximity ligation assay protein identities are encoded as DNA sequences by attaching DNA oligonucleotides to antibodies directed against the proteins of interest. Upon binding by pairs of antibodies to proteins present in the same molecular complexes, ligation reactions give rise to reporter DNA molecules that contain the combined sequence information from the two DNA strands. The ligation reactions also serve to incorporate a sample barcode in the reporter molecules to allow for direct comparison between pairs of samples. The samples are evaluated using a dual tag microarray where information is decoded, revealing which pairs of tags that have become joined. As a proof-of-concept we demonstrate that this approach can be used to detect a set of five proteins and their pairwise interactions both in cellular lysates and in fixed tissue culture cells. This paper provides a general strategy to analyze the extent of any pairwise interactions in large sets of molecules by decoding reporter DNA strands that identify the interacting molecules.

## Introduction

It is likely that future protein biomarkers will be selected from a far wider repertoire of molecular entities than those currently in focus. For example, it is of growing interest to distinguish protein isoforms that arise due to splicing, processing, or posttranslational modifications and that may have drastically different functions. Another emerging class of targets for protein analysis in basic research and to diagnose disease states and responses to therapy, are sets of interacting proteins, jointly executing various cellular functions [1]. Improved methods are therefore needed to search for proteins as well as their modifications and interactions, in order to investigate the predictive and diagnostic value of these molecular features in patient samples.

Most methods developed to study protein-protein interactions require genetic modification of proteins of interest to generate a detectable signal. The methods are therefore limited to research applications and cannot be applied for clinical samples. Immunofluorescence with two dye-labeled antibodies co-localizing as revealed via Förster resonance energy transfer (FRET) between the dyes can be used to analyze native proteins, however the method has a low signal to noise ratio, complicating analyses of patient samples. Moreover, with FRET only single interactions can be targeted in each analysis [2]–[4]. The VeraTag test is in use to investigate interactions among Her2 protein molecules in e.g. breast tumors, and it involves a pair of antibodies each linked to a fluorescent reporter and a photosensitizer molecule, respectively [5]. Upon photoactivation, the photosensitizer molecules cleave reporters in close proximity via the generated free radical oxygen. The liberated reporters are then recorded and used to quantify an average concentration of interacting target molecules in the sample. For multiplex studies of interactions among endogenous proteins the gold standard method has so far involved a combination of co-immunoprecipitation (Co-IP) followed by western blot or mass spectrometry, to look for interaction partners of a targeted protein. In order to carry out such experiments relatively large amounts of cells or tissues have to be lysed, disrupting cellular structures and local protein compartmentalization, potentially causing weakly interacting proteins to fall apart.

A method called interaction-dependent PCR (IDPCR) was recently developed by McGregor et al. [6] to detect interactions between ligands and targets in libraries of small molecules. The use of DNA barcodes overcame limitations in multiplexing for both bait and prey libraries and thus binary interactions between any combination of target and ligand could be detected in the same *in vitro* experiment. In the present report we have extended this concept to the analysis of interacting proteins by using proximity ligation for detecting and measuring interacting proteins.

The proximity ligation assay (PLA) [7] is an immunoassay utilizing so-called PLA probes – affinity reagents such as antibodies [8] modified with DNA oligonucleotides – for detecting and reporting the presence of proteins either in solution or *in situ* [9]. When two PLA probes bind the same or two interacting target molecules, the attached oligonucleotides are brought in close proximity. These oligonucleotides can then be joined by ligation to form an amplifiable reporter molecule. The requirement for recognition by two affinity reagents in proximity in order to generate a reporter molecule, followed by amplification by PCR or rolling-circle amplification (RCA), provides for highly sensitive assays to detect low amounts of proteins. The assays can also be used to study interactions among proteins [9]–[11] or between protein and DNA sequences [12], [13]. PLA has been used to detect potential biomarkers in plasma, serum, cerebrospinal fluid, and cell lysates, both single proteins, protein aggregates (Aβ protofibrils) [14], and interacting proteins have been targeted [10], [11]. *In situ* PLA is a variant of the technique that permits visualization of the location of proteins, protein-protein interactions, and secondary modifications such as phosphorylations [15] and glycosylations [16] in fixed cells and tissues without a need for genetic modification of the investigated cells.

PLA has the potential to simultaneously assess large numbers of proteins and protein-protein interactions in a sample, since tag sequences can be introduced in the synthetic reporter DNA molecules, and several methods are available for detecting numerous DNA sequences simultaneously. Solution-phase PLA has been adapted for multiplex analyses of proteins with readout, either by quantitative PCR [17]–[19] or using next generation DNA sequencing [20].

Here we have combined PLA with readout via dual tag microarrays (DTM) [21] for parallel, sensitive detection of sets of proteins and protein-protein interactions. Using the DTM technique each microarray feature specifically detects and amplifies signals from reporter nucleic acid molecules that comprise two tag sequence elements, representing the interacting proteins. We have previously used the DTM technique to measure cDNA levels as well as reporter molecules from proximity ligation and padlock probe assays, achieving a radically decreased risk of cross-hybridization compared to standard microarray approaches [21]. Here we apply PLA with DTM readout to identify pairs of DNA tags, derived from the two PLA probes whose DNA strands have become joined by ligation upon coordinated binding to the same target protein or protein complex. The DTM readout can be applied for analysis of PLA products reflecting protein levels and interactions both in liquid samples and for fixed tissue sections on slides, to provide measures of all binary combinations of PLA probes. The approach can be generalized to interrogate all interactions within larger sets of investigated proteins. It has previously been shown that dual color microarray analysis of proteins, where two samples are compared directly against each other, outperforms single color approaches when it comes to reproducibility and power of discrimination of the assay [22]. We therefore designed a cassette connector oligonucleotide to facilitate sample barcoding for dual-color readout of results from analyses of pairs of samples.

**Figure 1:**
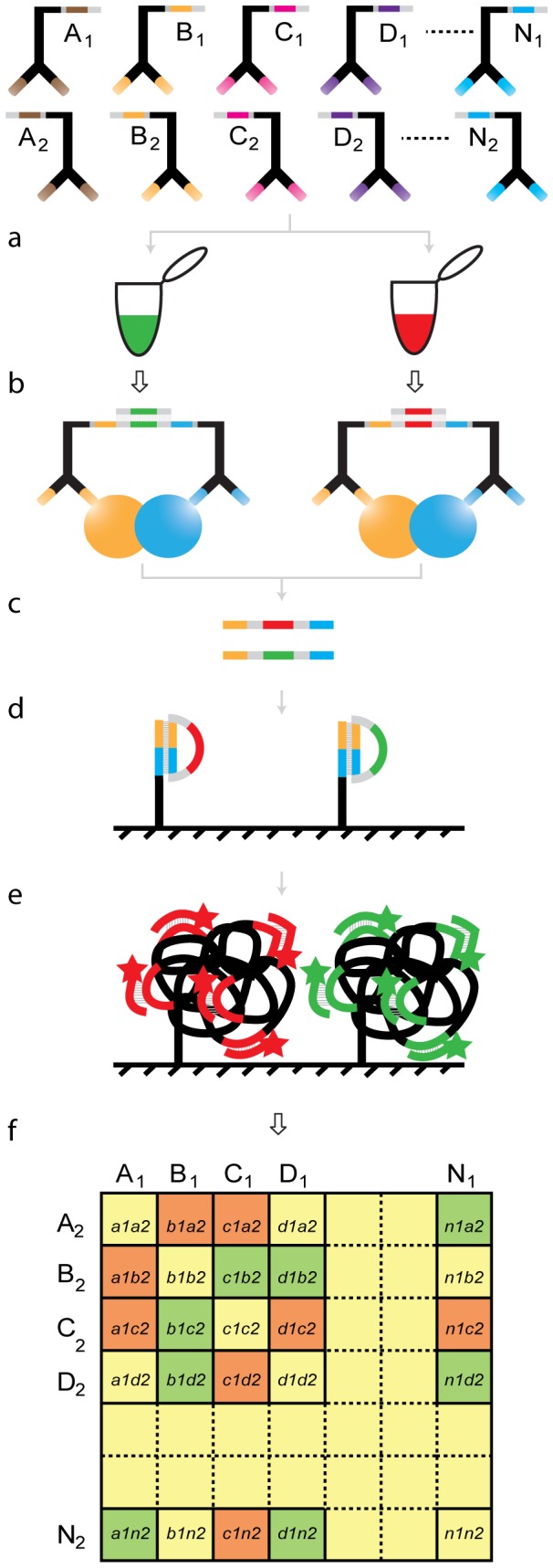
Schematic illustration of PLA analysis with DTM readout. **a**) The PLA probes, each carrying unique DNA sequences (A1, B1, …, N1, or A2, B2, …, N2) that code for their target antigens, are incubated with the samples to be examined. **b**) The oligonucleotides on pairs of PLA probes that have bound their targets in close proximity are ligated to give rise to reporter DNA strands. In the ligation step a sample barcode is introduced in the ligation product to allow for dual color comparisons of results for two samples in the same array spot. **c**) The ligation products are amplified by PCR and treated with DNA modifying enzymes to generate single stranded reporter molecules with barcodes identifying the targeted proteins at both ends. **d**) The reporter molecules are hybridized to oligonucleotides complementary to pairs of protein tags (e.g. N1N2), on a microarray, thereby allowing the reporter strands to be ligated into circles**. e**) The circularized reporter molecules finally template RCA, primed by the oligonucleotides on the array, and the RCA products are detected by hybridization with Cy3 or Cy5 labeled detection oligonucleotides. **f**) The Cy3 and Cy5 intensities are measured, and the ratios between the two colors are analyzed for each feature to detect differences in interaction patterns and protein abundances between the two samples.

The combination of PLA with dual color DTM readout for multiplex detection of proteins and protein-protein interactions is illustrated in Figure 1. The assay is designed for direct comparison of each potential protein interaction in a set of investigated proteins between two samples in the same spot of a microarray using dual color readout. PLA is performed either on immune-precipitates of molecules of interest from cell lysates, or on cells fixed on a microscope slide, using a variant of methods previously described [10], [23] (Figure 1a). Reaction products from individual samples are barcoded with a unique DNA sequence by interposing a short sample-specific sequence when pairs of PLA probes are joined by ligation (Figure 1b). Thereafter the ligated reporter molecules from the two samples to be compared are pooled into one reaction tube and jointly amplified by PCR (Figure 1c). The amplified reporter molecules are allowed to hybridize to the arrays (Figure 1d). A ligase is subsequently added, allowing only the reporter with two barcode tags complementary to the array oligonucleotide to be ligated into circles. The circles will template localized DNA amplification by RCA on the microarray, as previously described [21]. Detection oligonucleotides, specific for the two sample barcodes and labeled with Cy3 or Cy5, are added (Figure 1e). The recorded fluorescence ratio of the two colors in each spot provides a measure of the difference in levels of proteins or protein interactions between the two samples (Figure 1f).

To our knowledge this is the first description of an immunoassay capable of detecting all possible binary interactions among a given set of proteins in biological samples. We demonstrate analyses of protein interactions both in cell lysates and in cells fixed onto glass slides, with readout using binary microarrays.

## Results

**Figure 2:**
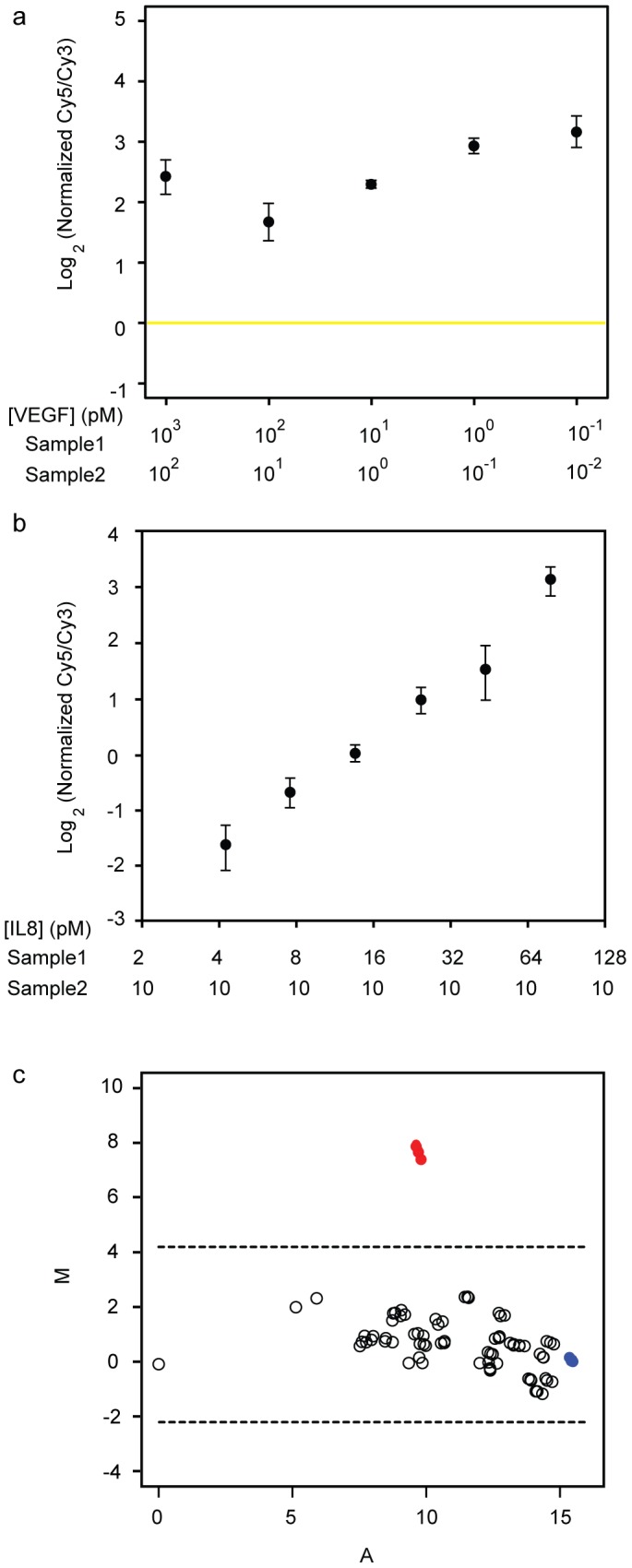
Analysis of the concentration range over which differences of protein concentrations can be recorded. **a**) 10-fold differences in concentration of VEGF between two samples were successfully detected even when the concentrations were as low as 100 and 10 fM. For normalization purposes IL8 was spiked into all samples at a fixed concentration of 10 pM, and the fluorescence ratio for VEGF was normalized against the fluorescence ratio for IL8. **b**) Two-fold differences of IL8 concentrations were readily detected. Plotted are the fluorescence ratios when comparing a sample with the indicated concentration of IL8 and a sample with 10 pM IL8, after normalization for the fluorescence signal from detection of 10 pM VEGF. **c**) Detection of 10 pM VEGF over background in cell lysates. Two samples – cell lysate with or without VEGF spiked in – were compared. The results are plotted in an MA-plot with the log_2_ ratios between Cy5 and Cy3 (M = log_2_(Cy5/Cy3)) on the y-axis and the log_2_ average intensity of Cy5 and Cy3 (A = 0.5 log_2_(Cy5×Cy3)) on the x-axis. Red, filled circles show triplicate measurements of VEGF. Blue, filled circles represents triplicate measures of a spiked in oligonucleotide control. The remaining unfilled circles represent the measurements of the other four targeted proteins (p50, RelA, RelB, IκBα) and all their possible pairwise interactions. Dotted lines indicate the 95% confidence interval (2 standard deviations from the mean ratio between Cy5 and Cy3).

Proximity ligation using the SP-PLA technique provides opportunities to investigate interactions among proteins in cells and tissues. Here we have optimized the SP-PLA protocol for analyzing the levels of all pairwise protein interactions in a set of targeted proteins, with microarray readout as presented in the supplementary material (File S1, and Figure S1, Figure S2, Figure S3). To benchmark the protocol and to establish its performance for protein detection we first analyzed the presence of VEGF and IL8, spiked into buffer, before continuing to detect protein interactions in more complex samples. Proximity ligation was performed on two samples, labeling reaction products with sample-specific sequences. Products from pairs of samples were pooled prior to PCR and DTM readout, where products from the two samples were detected in the same array features using probes labeled with distinct fluorophores. The purpose of the experiment was to investigate differences in protein abundance between two samples in the presence of another protein maintained at the same level in all samples. The concentration of VEGF was varied over a broad range in order to investigate the dynamic range of the assay, while always maintaining a constant ten-fold different concentration of VEGF between the two samples to be pairwise compared. The highest VEGF concentrations used were 1 nM/10 nM, and the lowest 10 fM/100 fM. In addition, all samples contained the reference protein IL8 at a constant concentration of 10 pM. Figure 2a presents the fluorescence ratio for VEGF normalized by the fluorescence ratio for IL8. The experiment demonstrates that the ten-fold difference in VEGF concentration between the two samples could be detected over a broad range of concentrations from ∼10 nM down to ∼100 fM, with IL8 as a constant reference in both samples.

To next investigate the precision of the assay, two-fold dilutions of IL8 in PLA buffer were analyzed, along with 10 pM of VEGF spiked into each sample as PLA controls and for normalization. These samples were all individually compared with a sample of 10 pM IL8 and 10 pM VEGF spiked into PLA buffer. Figure 2b shows that the assay is able to distinguish concentration differences of the investigated protein of less than two-fold over the full range.

Next, we investigated if changes in protein concentration also could be measured in realistic biological samples, and for a greater number of targeted proteins. We prepared cell lysates from cells overexpressing members of the well-studied NFκB family of proteins, namely p50, RelA, RelB, and IκBα. The lysates were split in two aliquots and VEGF was spiked at a concentration of 10 pM into one of them. The pairs of samples were compared in an assay with PLA probes present for detection of all five proteins. Figure 2c presents the results in an MA-plot where the average intensity for a protein is plotted against the ratio between the two colors detected in the array. This illustrates the average of the protein concentration in both samples (A; x-axis) versus the difference in protein concentration between the two samples (M; y-axis), clearly showing that VEGF gives an average signal in the middle of the range of protein concentrations but that higher signals were detected for the sample with added VEGF as compared to the sample where no VEGF was spiked in. Accordingly, the assays distinctly revealed differences in VEGF concentrations between the two samples also in the complex context of cellular lysates.

We used PLA with DTM readout for assessing pairwise protein interactions among the NFκB subunits RelA, RelB, and p50. It is well known that all these proteins can dimerize with each other both as homo- and heterodimers, and that the biological functions of these dimers can be prevented by forming larger complexes with the inhibitory IκB proteins (e.g. IκBα, reviewed in Hoffman et al. 2007 [24]). As a proof of concept we therefore investigated all six possible binary interactions between these four proteins. We also included reagents for detecting a housekeeping protein (glyceraldehydes 3-phosphate dehydrogenase; GAPDH) in order to normalize for any differences in sample input, and we thus investigated ten possible binary interactions in total.

As a first measure we developed an antibody validation pipeline (Figure S4) to ensure that the antibodies we used could recognize the target protein in native form also while in complex with its known interaction partners and in such a manner that each protein target could first be captured using an immobilized antibody, followed by the addition of two PLA probes. This was validated by (i) western blot analyses of lysates from cells transfected with the target protein in comparison to wild type lysates, (ii) Co-IP read out by western blot and (iii) SP-PLA with different combinations of antibodies targeting the same protein. Since SP-PLA requires the antigen to be captured and then bound by at least two PLA probes, in total at least three epitopes must be available for simultaneous binding on the same target molecule or pairs of molecules. Therefore, in some cases antibodies raised against different parts of the protein had to be combined to generate functional SP-PLAs. In the validation pipeline we have disregarded the fact that some of the target proteins may be present as homodimers. As a result of this we cannot tell to what extent the detection of protein content reflects the total protein concentration or that of homodimers. The specificity of the antibodies was also evaluated to confirm detection of the proper target molecules. Moreover, we only employed antibodies that generated the same low signals in cell lysates whether the cells had been transfected with any other NFκB family member or not (data not shown).

**Figure 3:**
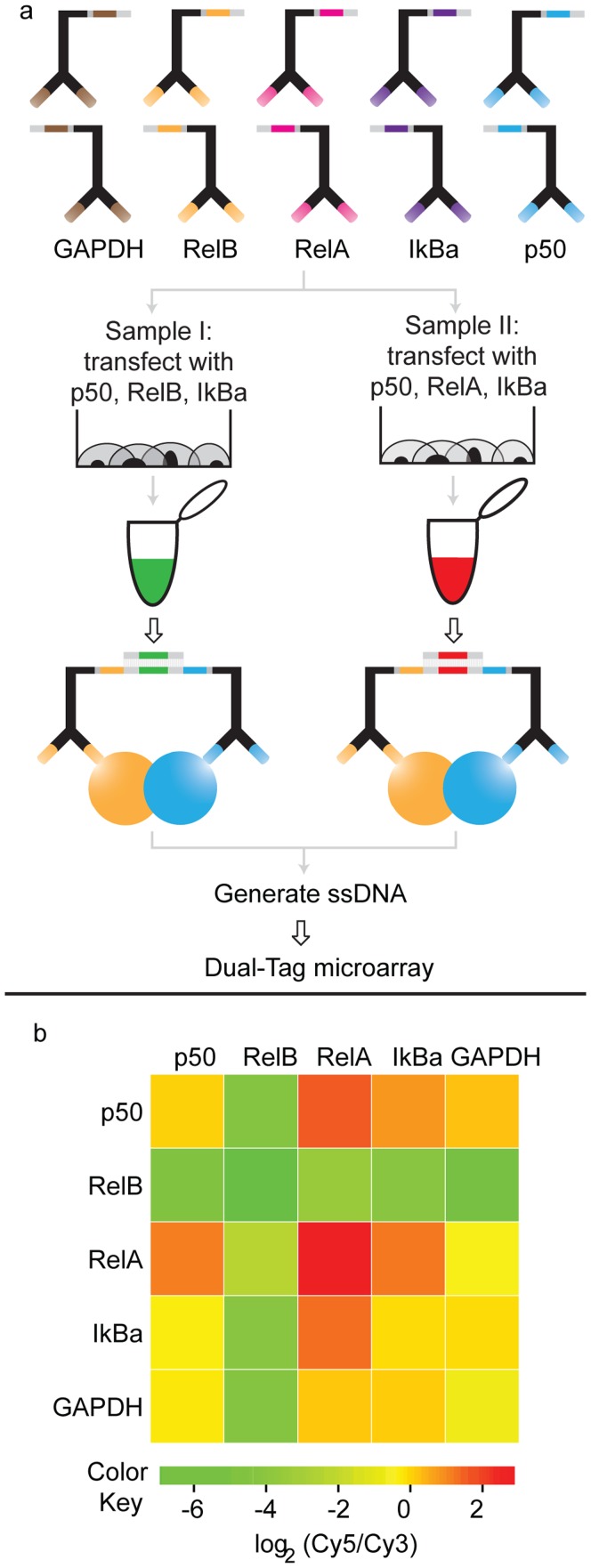
Detection of p50, RelA, RelB, IκBα and GAPDH, and any pairwise interactions in cell lysates. **a**) Cells were transfected with genes encoding proteins of the NFκB family; either p50, IκBα and RelB (sample I); or p50, IκBα and RelA (sample II). The two populations of cells were lysed and the PLA probes were added to the cell lysates in separate reactions. After generating the reporter molecules by ligation, the two samples were pooled to amplify the ligation products by PCR, followed by readout via DTM. **b**) The results are represented in a heat map where negative log_2_ Cy5/Cy3 ratios (color coded as more green) indicate a higher abundance of proteins or protein interactions in sample I than in sample II, while positive log_2_ Cy5/Cy3 ratios (color coded as more red) indicate the opposite situation.

The targeted proteins of the NFκB family were transiently overexpressed and we analyzed differences in levels of proteins and of interacting proteins between two samples where either RelA or RelB was omitted from the transfections. Our results demonstrate that we can detect a difference in the abundance of the two proteins, and we could also demonstrate the relative levels of interactions (Figure 3, and Table S1a). In the experiment we used lysates from cells that had been transiently transfected with constructs encoding the NFκB proteins. We compared two lysates, one where the cells had been transiently transfected with RelA, p50 and IκBα and another transfected with the same proteins but replacing RelA with RelB. As expected we detected a greater abundance of RelB and of RelB in its known interaction with p50 in samples transfected with RelB as compared to samples transfected with RelA. Conversely, in samples transfected with RelA we saw a greater abundance of RelA and RelA interacting with its known partners p50 and IκBα as compared to the sample transfected with RelB. Similar results were observed when the experiment was replicated using the opposite sample barcodes for the two samples (Figure S5).

**Figure 4:**
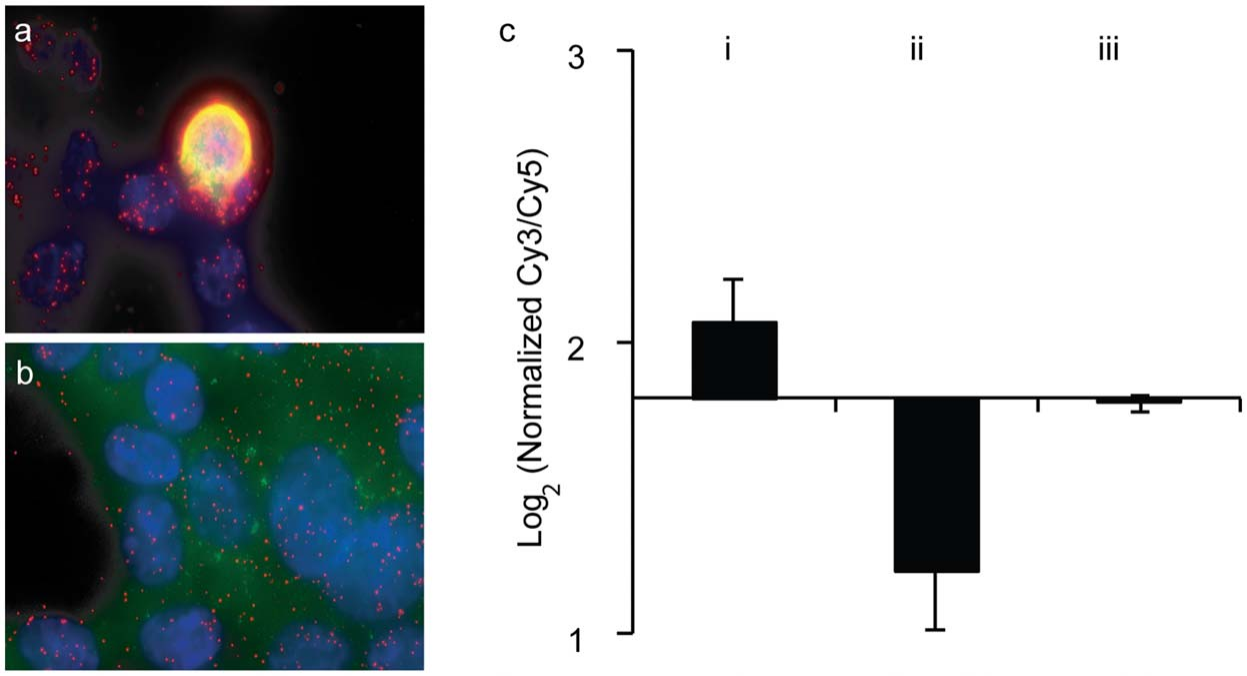
DTM readout of PLA on fixed cells. *In situ* PLA (red) and immunofluorescence (green) detection of SMAD4 in **a**) cells transiently transfected with SMAD4 and **b**) wt cells having only endogenous levels of SMAD4. **c**) DTM readout of the ratios between SMAD4 from transfected cells and wt cells: transfected/wt (i), wt/transfected (a dye-swap experiment) (ii), transfected/transfected (iii), normalized against the ratio of beta-actin.

To demonstrate the possibility to use microarray for analysis of PLA reactions performed *in situ* we designed an oligonucleotide system for readout either by DTM or *in situ* PLA with RCA. The oligonucleotides conjugated to antibodies were constructed to template ligation of two separate connector oligonucleotides to form a DNA circle when pairs of PLA probes co-localize by binding the same protein or two interacting proteins. One of the connector oligonucleotides comprises two PCR primer sites, and the other encodes the two tags for correct localization on the oligonucleotide arrays, and one of two possible sample tags used for fluorescent detection of RCA products generated in the array features. To demonstrate the performance of the method, we investigated levels of the SMAD4 protein in transfected and untransfected cells (Figure 4, experimental design described in greater detail in Figure S6). After ligation with different connector oligonucleotides for the two samples, the DNA circles were detected either by *in situ* PLA with RCA in cells prepared for microscopy, or by PCR amplification and then DTM readout. For *in situ* PLA detection (Figure 4a, b) phi 29 polymerase was then added to initiate RCA, followed by decoration of the amplification products by hybridization with fluorescent oligonucleotides. Relatively high endogenous SMAD4 expression was detected in the cells, while as illustrated by the single transfected cell in Figure 4a, only a minor proportion of the cells expressed the transgene.

Microarray analysis of ligation products was performed after first releasing ligated circular DNA from transfected and untransfected cells through the addition of proteinase K. The ratio of the two fluorophores thus reflects the relative SMAD4 abundance in the two cell populations. β­actin protein was detected in the same reactions for normalization. Figure 4c shows the SMAD4 expression ratios for transfected versus untransfected cells, including dye-switch controls. As expected, PLA with DTM readout revealed that transfected cells expressed greater amounts of SMAD4 compared to untransfected cells.

## Discussion

We have previously demonstrated that PLA offers highly sensitive and specific detection of proteins and their interactions over wide dynamic ranges either in solution or *in situ*, and the method can be applied either for transfected or normally expressed target proteins. Interactions are dynamic processes that are preferentially detected in their natural environment. In this paper we have established a scalable method using microarrays for recording levels of proteins and all pairwise interactions among a targeted set of proteins. The method has been applied for investigating four proteins of the NFκB family along with one housekeeping protein. We demonstrate that the approach can be adapted to different sample types by analyzing both cell lysates and fixed cell samples, and the assays should thus be suitable for analysis of clinical samples such as plasma, serum or other bodily fluids, as well as fixed cells or tissue sections. By using DNA barcodes to represent the targeted proteins, and taking advantage of efficient techniques for nucleic acid analysis, the method allows analysis of the entire interaction space among the set of targeted proteins. This opens up unique possibilities for studying all possible interactions and posttranslational modifications among large groups of proteins, either for research or diagnostic purposes. Also larger complexes of interacting proteins can be investigated by identifying pairwise interactions.

Both quantitative real time PCR and next generation sequencing have previously been used to read out multiplex PLA experiments [17]–[20]. Recently, Lundberg et al [19] developed four 24-plex PLA reactions, using a microfluidic system from Fluidigm to conveniently run real time PCR in chips with 96 amplification reactions for each of 96 DNA samples per chip. Since the potential for binary interaction analysis scales as the square of the number of investigated targets, DTM offers an attractive alternative for inexpensive high-throughput readout. By using two-color DTM analyses, distinctly bar-coded PLA ligation products from pairs of samples can be pooled prior to PCR amplification, allowing the ratios for the corresponding analytes in the two samples to be determined even when array features are saturated, thus achieving a broad dynamic range. The proposed method has limitations for absolute quantification of the abundance of proteins or protein-protein interactions, but the method is excellently suited to screen for quantitative differences between pairs of samples.

It could be of great value to use the method to screen for differences in levels of proteins and their binary interactions between e.g. cancer and normal tissue, and to follow this by qualitative *in situ* PLA analyses of the individual or interacting proteins using the same reagents. A main rate limiting factor for further multiplexing of the assay will be the identification of high quality affinity reagents. The quality of the antibodies strongly influences both the sensitivity and the specificity of the assays. We have applied a strict validation pipeline for all binders used in the assays shown herein, to ensure that the antibodies are specific and do not compete with the binding sites for the targeted protein interactions.In conclusion, the PLA-DTM provides a scalable approach for investigating an extended range of molecular features and potential biomarkers in different types of biological samples. We believe the potential of our method to assess a broad range of pairwise interactions can have important applications in numerous biological, medical and biotechnological contexts. Next generation sequencing offers greatly enhanced opportunities for using PLA to decode patterns of protein interaction in normal physiology and in pathological lesions.

## Materials and Methods

### Solid Phase PLA

SP-PLA was performed as previously described [23] with the following modifications. After incubation with 1 nM PLA probes, 50 µl ligation mixture was added containing 10 mM Tris-HAc, 10 mM MgAc, 50 mM KAc, 0.5 U T4 DNA ligase (Fermentas), 0.1 µM of either one of the two connector oligonucleotides preincubated with a three-fold excess of its cassette oligonucleotide (Table S2) and 0.08 mM ATP (Fermentas). The reaction was carried out for 5 min at room temperature (RT).

After ligation the reactions were washed twice and thereafter treated with 30 µl exonuclease mixture (50 mM KAc, 20 mM Tris-HAc pH 7.5, 3 mM MgAc, 1 mM DTT (Invitrogen), 5 U lambda exonuclease (New England Biolabs), 10 U phi29 DNA polymerase (Fermentas) and 0.05 µg/µl BSA (New England Biolabs)) for 30 min at RT. After two washes 15 µl of two reactions to be compared were pooled for joint analysis. PCR was performed in a total volume of 50 µl containing 50 mM KAc, 20 mM Tris-HAc pH 7.5, 3 mM MgAc, 1 mM DTT, 0.2 mM dNTPs containing dUTP instead of dTTP (Fermentas), 0.1 U uracil-DNA glycosylase (UDG; Fermentas), 1.5 U platinum Taq polymerase (Invitrogen) and 100 nM of forward and reverse universal PCR primers (Table S2). Amplification was carried out by incubating at 95°C for 2 min, followed by 20 cycles of 95°C for 15 sec and 60°C for 1 min.

### *In situ* PLA

*In situ* PLA was performed as previously described [9]. Briefly, cells were cultured on BD Falcon™ 8-wells culture slides (BD NJ USA) and fixed with 3% paraformaldehyde (Sigma) in phosphate-buffered saline (PBS) for 30 min at RT and permeabilized by treatment with 0.05% Triton in PBS. Chambers were removed and the areas with cells were demarcated using a hydrophobic pen (DAKO Cytomation) to establish separated reaction wells on the slide. Slides were then blocked with *in situ* blocking buffer (20% human serum (Jackson ImmunoResearch), 100 µg/ml salmon sperm DNA (Invitrogen), 5 mM EDTA and 0.05% Tween-20 in PBS) for 2 h at RT, followed by washing for twice, 2 min in PBS, 0.05% Tween-20 at RT.

The PLA probes (Table S3) were added in 40 µl *in situ* blocking buffer supplemented with 2.5 mM cystein (Sigma), followed by 5 min washing in 10 mM Tris-HCl pH 7.5, 0.05% Tween-20 and then 2×2 min in Tris-buffered saline (TBS), 0.05% Tween-20 at RT.

PLA probes were ligated by adding 125 nM of each connector and backbone oligonucleotide (Table S2), 10 mM Tris-HAc (pH 7.5), 10 mM MgAc, 50 mM KAc, 0.05 U/µl T4 DNA ligase, 250 mM NaCl, 0.25 µg/µl BSA and 0.05% Tween-20 to the slides and incubating at 37°C. Ligation reactions were stopped after 30 min, followed by 2×2 min washes in TBS with 0.05% Tween-20 at RT.

### Transfer of Ligation Products from in situ Slides to PCR

For DTM readout, ligation products were released from the slides by adding 1 U proteinase-K (Fermentas) in 40 µl 50 mM KAc, 3 mM MgAc, 20 mM Tris-HAc and 1 mM DTT to each well on the culture slide, followed by incubation at 37°C for 1 h. Ligation products were transferred to a reaction tube and the protease was inactivated at 95°C for 20 min. After inactivation, 5 µl of ligation products from transfected cells and wildtype (wt) cells, respectively, were pooled pair-wise, mixed with 40 µl PCR mix (details above), and amplified by PCR for 7 cycles of 95°C for 15 sec and 60°C for 1 min. Ten µl of PCR products was used for microarray readout.

### Translation of Molecular Interactions into a Reporter Molecule Library

Before microarray readout, the Taq polymerase in the amplification reactions was inactivated by adding 1 U proteinase-K to 25 µl PCR product mix. Incubation at 37°C for 1 h was followed by denaturation of the protease at 95°C for 20 min. Next, 10 U each of the restriction enzymes MboI (Fermentas) and NlaIII (New England Biolabs) were added to the proteinase-K-treated PCR products along with 0.4 µg/µl BSA, followed by incubation at 37°C for 2 h. After restriction digestion, the uracil residues in the amplified and digested fragments were degraded by adding 1 U UDG to each reaction, incubating at 37°C for 30 min, and then heating to 95°C for 20 min.

### DTM Readout

DTM readout was performed essentially as previously described [21]. Briefly, to each reaction chamber on the microarray (pre-heated to 55°C) 50 µl ligation mixture was added, comprising 10 µl pre-treated PCR product at a final buffer concentration of 20 mM Tris-HCl pH 8.3, 35 mM KCl, 7.2 mM MgCl2, 3 mM NAD, 0.1% Triton X100, 0.05% BSA and 5 U Ampligase (Epicentre). The ligation reactions were incubated at 55°C for 1 h. The individual wells were washed with 1 ml 1× TNT buffer (100 mM Tris pH 7.5, 150 mM NaCl, 0.05% Tween 20), and the array was dissembled to wash the whole glass slide with 0.1× SSC (15 mM NaCl, 1.5 mM Na-citrate). Next, each microarray chamber received 50 µl RCA mixture comprising 33 mM Tris-HAc pH 7.9, 10 U phi29 polymerase, 0.5 µg/µl BSA, 200 nM dNTP, 10 mM MgAc, 66 mM KAc, 1% Tween-20, and 1 mM DTT, and incubated at 37°C for 30 min. The silicon mask was removed and the array washed with 1× TNT buffer and rinsed with 0.1× SSC. After reassembling the reaction chamber to the array, 10 nM of each detection oligonucleotide (Table S2) were hybridized to the RCA products in array hybridization buffer at 55°C for 1 h. The arrays were washed in 1× TNT buffer and then in 0.1× SSC at 46°C for 15 min. Finally, the arrays were dried by centrifugation and they were scanned by a GenePix 4000B microarray scanner and processed by GenePix Pro 6.0 software. The fluorescence intensities are mean values for individual microarray features. The ratios of Cy5 and Cy3 were mean ratios of fluorescence intensities of triplicate array features. The normalization values were calculated by:
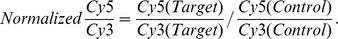



Heatmaps were generated by R 2.10.1/BioConductor algorithms.

## Supporting Information

Figure S1 **Schematic illustration of PLA analysis with DTM readout.** **a)** The PLA probes are incubated with the samples to be examined. **b)** The oligonucleotides on pairs of PLA probes that have bound their targets in close proximity are ligated pairwise to give rise to reporter DNA strands. In the ligation step a sample barcode is introduced in the ligation product via a cassette connector oligonucleotide to allow for dual color comparisons of results for two samples in the same array spots. **c)** The ligation products are treated with exonucleases prior to **d)** pooling of pairs of samples to be compared against each other, followed by amplification of the ligation products with PCR. **e)** The PCR products are then treated with restriction enzymes and **f)** UDG, to generate single stranded reporter molecules with barcodes identifying the targeted proteins at both ends. **g)** The reporter molecules are hybridized to oligonucleotides complementary to pairs of protein tags, on a microarray, thereby allowing the reporter strands to be ligated into circles**. h)** The circularized reporter DNA molecules finally template RCA, primed by the oligonucleotides on the array, and the RCA products are detected by hybridization with Cy3 or Cy5 labeled detection oligonucleotides, depending on which sample tag was introduced in the reporter DNA circle.
(TIF)

Figure S2 **Optimization of the generation of reporter DNA strands. a)** The assay background was significantly reduced by treating the ligation products with exonucleases and optimizing the number of PCR cycles. Two individual PLA reactions for detection of 10 pM VEGF were carried out with oligonucleotide system A1A2 and C1C2. After ligation, the ligation products were treated with a 50 µl exonuclease mix containing 0.1 U/µl lambda exonuclease and 0.2 U/µl phi29 polymerase, 0.01 µg/µl BSA in 1× special buffer (50 mM KAc, 3 mM MgAc, 20 mM Tris-HAc pH 7.5, 1 mM DTT) at 37°C for 30 minutes, followed by washing in 1× PBS, 0.05% Tween20. The treated products were pooled and amplified by PCR. Untreated ligation products were pooled and used as assay controls. The PCR-amplified products were diluted according to the results from analytical realtime-PCR quantification with specific primer pair combinations: AC and CC. Signals generated from primer combination AC are defined as background, while signals generated from primer combination CC are defined as signals. **b)** The effect of the Taq polymerase was investigated by addition of proteinase K at different points after PCR in comparison to reactions where no proteinase K was added. **c)** The buffer was optimized so that all enzymatic reactions could be performed in the same buffer. VEGF detection by PLA performing ligation and PCR amplification in the standard PCR buffer and the special buffer designed for the assay. **d)** The enzymatic efficiencies in the two separate buffers were investigated by treating PCR-products with the restriction enzymes NlaIII and MboI and UDG.
(TIF)

Figure S3 **Comparison of signal from matched or partially mismatched reporter strands on DTM.** The signal from the array spot with both tags complementary to the single stranded ligation product is significantly greater than for array features where only one of the tags is complementary.
(TIF)

Figure S4 **Antibody validation pipeline.** All antibodies used for detection of NFκB-family proteins were validated by western blot to investigate if they could bind a protein of the estimated size, Co-IP to confirm that they could bind the native protein while interacting with its known interaction partners, and SP-PLA to find the best combination of binders to be used for PLA. **a)** Immunoprecipitation with antibodies against p50, RelA and RelB showed that the known interaction partners were co-immunoprecipitated together with the targeted protein. **b)** Different antibodies were used for capture (c), PLA probe 1 (p1) and probe 2 (p2) in SP-PLA. The combination with A301–823A as capture and one of the probes together with ab7970 as the other probe was superior in generating a high signal over background for detection of RelA when compared to using A301–823A as capture and ab7970 as the two PLA probes.
(TIF)

Figure S5 **Validation of detection of protein-interaction between the NFκB-family proteins.** The detection of protein interactions among the NFκB-family proteins was repeated with the two sample tag sequences, introduced during the ligation reaction, switched for the two samples. In this heat map negative log_2_ Cy5/Cy3 ratios (green) indicate a greater abundance in sample I (transfected with p50, RelA and IκBα), while positive log2 Cy5/Cy3 ratios (red) indicate a greater abundance in sample II (transfected with p50, RelB and IκBα). Raw data are presented in Table S1b.
(TIF)

Figure S6 **Experimental setup for** ***in situ*** **PLA detection of Smad4 with fluorescent** ***in situ*** **detection and DTM readout.** PLA probes directed to Smad4 and beta-actin were applied to slides with fixed cells that had or had not been transfected with Smad4. Connector oligonucleotides were added and joined into circular reporter molecules by enzymatic ligation. The reporter DNA circles were detected *in situ* in cells fixed on slides by allowing them to guide rolling circle amplification to generate large concatemers of DNA. The amplification products were then detected by hybridization with a fluorescence labeled detection oligonucleotide. For an alternative readout on microarrays, reporter DNA circles were released from slides by treatment with proteinase K, amplified by PCR and then read out according to the protocol for DTM readout.
(TIF)

Table S1 **Raw data for** **Figure 3** **and Figure S5.** Listed are the measured Cy5-intensities and Cy3-intensities for the experiments presented in **a)** Figure 3 and **b)** Figure S5. The ratios between the Log_2_(Cy5/Cy3) are calculated for individual array features, and an average value is calculated for the three replicate features within a subarray.
(TIF)

Table S2 **List of oligonucleotides used in the experiments.** Listed are the sequences of all oligonucleotides used in the experiments together with their modifications in the 3′-free and 5′-free ends respectively.
(TIF)

Table S3 **List of antibodies used in the experiments.** Listed are the antibodies with their target proteins, catalog number and manufacturer, any modification, type of immunoglobulin (Ig), and concentrations in various assays.
(TIF)

File S1 **Supplementary Results and Materials and methods.**
(PDF)
