# Correction: Profiling Cellular Protein Complexes by Proximity Ligation with Dual Tag Microarray Readout

**DOI:** 10.1371/journal.pone.0119890

**Published:** 2015-03-25

**Authors:** The PLOS ONE Staff

Four oligonucleotide sequences are missing from S2 Table of the published article. Please view the correct S2 Table here.

## Supporting Information

S2 Table List of oligonucleotides used in the experiments. Listed are the sequences of all oligonucleotides used in the experiments together with their modifications in the 3′-free and 5′-free ends respectively.
(TIF)
