# Supplementary figures and images for: Profiling Cellular Protein Complexes by Proximity Ligation with Dual Tag Microarray Readout
Source: PLoS One. 2012 Jul 10;7(7):e40405. doi: 10.1371/journal.pone.0040405 (PMC3393744; doi:10.1371/journal.pone.0040405)

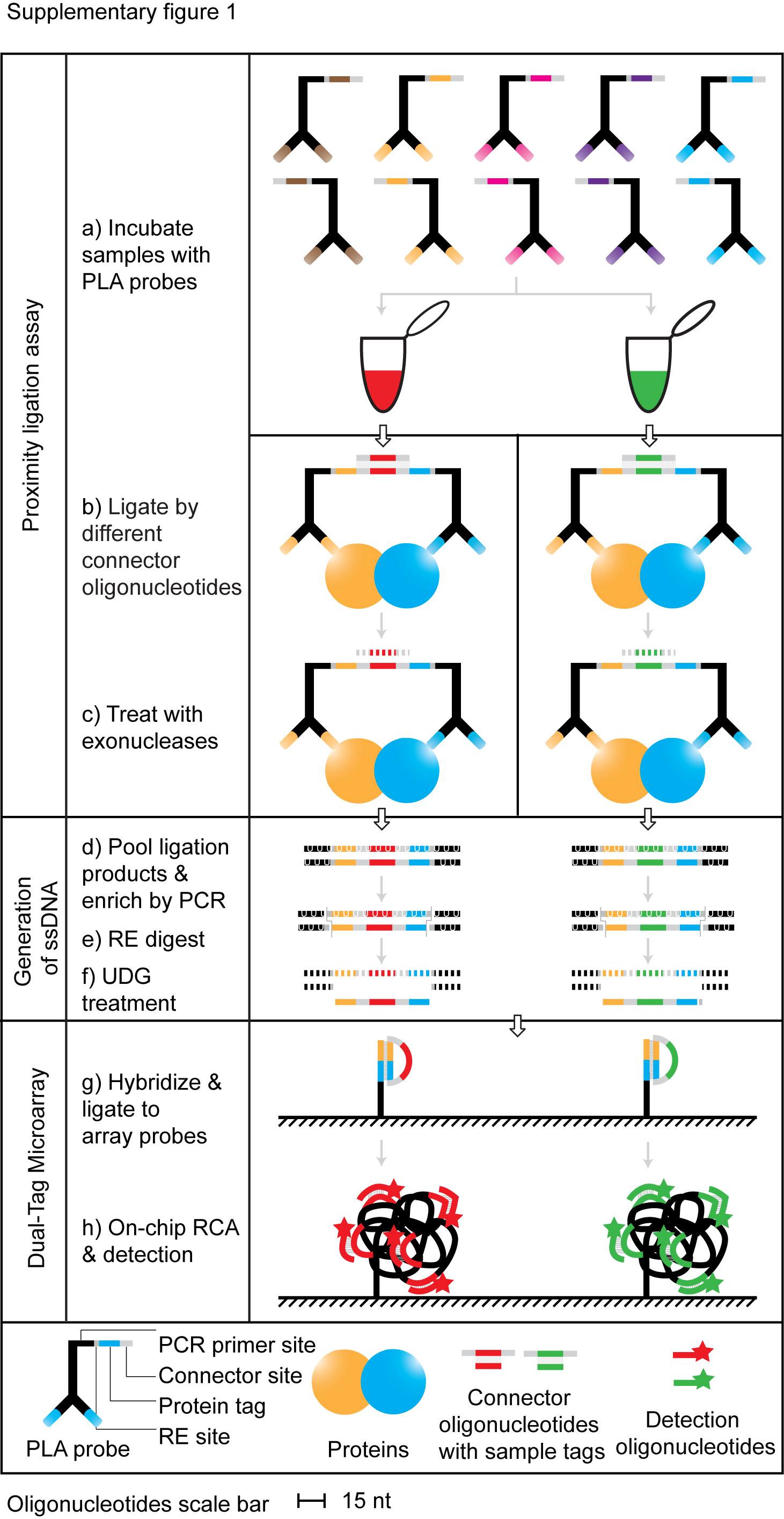

Supplement: Figure S1 — Schematic illustration of PLA analysis with DTM readout. a) The PLA probes are incubated with the samples to be examined. b) The oligonucleotides on pairs of PLA probes that have bound their targets in close proximity are ligated pairwise to give rise to reporter DNA strands. In the ligation step a sample barcode is introduced in the ligation product via a cassette connector oligonucleotide to allow for dual color comparisons of results for two samples in the same array spots. c) The ligation products are treated with exonucleases prior to d) pooling of pairs of samples to be compared against each other, followed by amplification of the ligation products with PCR. e) The PCR products are then treated with restriction enzymes and f) UDG, to generate single stranded reporter molecules with barcodes identifying the targeted proteins at both ends. g) The reporter molecules are hybridized to oligonucleotides complementary to pairs of protein tags, on a microarray, thereby allowing the reporter strands to be ligated into circles. h) The circularized reporter DNA molecules finally template RCA, primed by the oligonucleotides on the array, and the RCA products are detected by hybridization with Cy3 or Cy5 labeled detection oligonucleotides, depending on which sample tag was introduced in the reporter DNA circle. (TIF) [file pone.0040405.s001.tif]

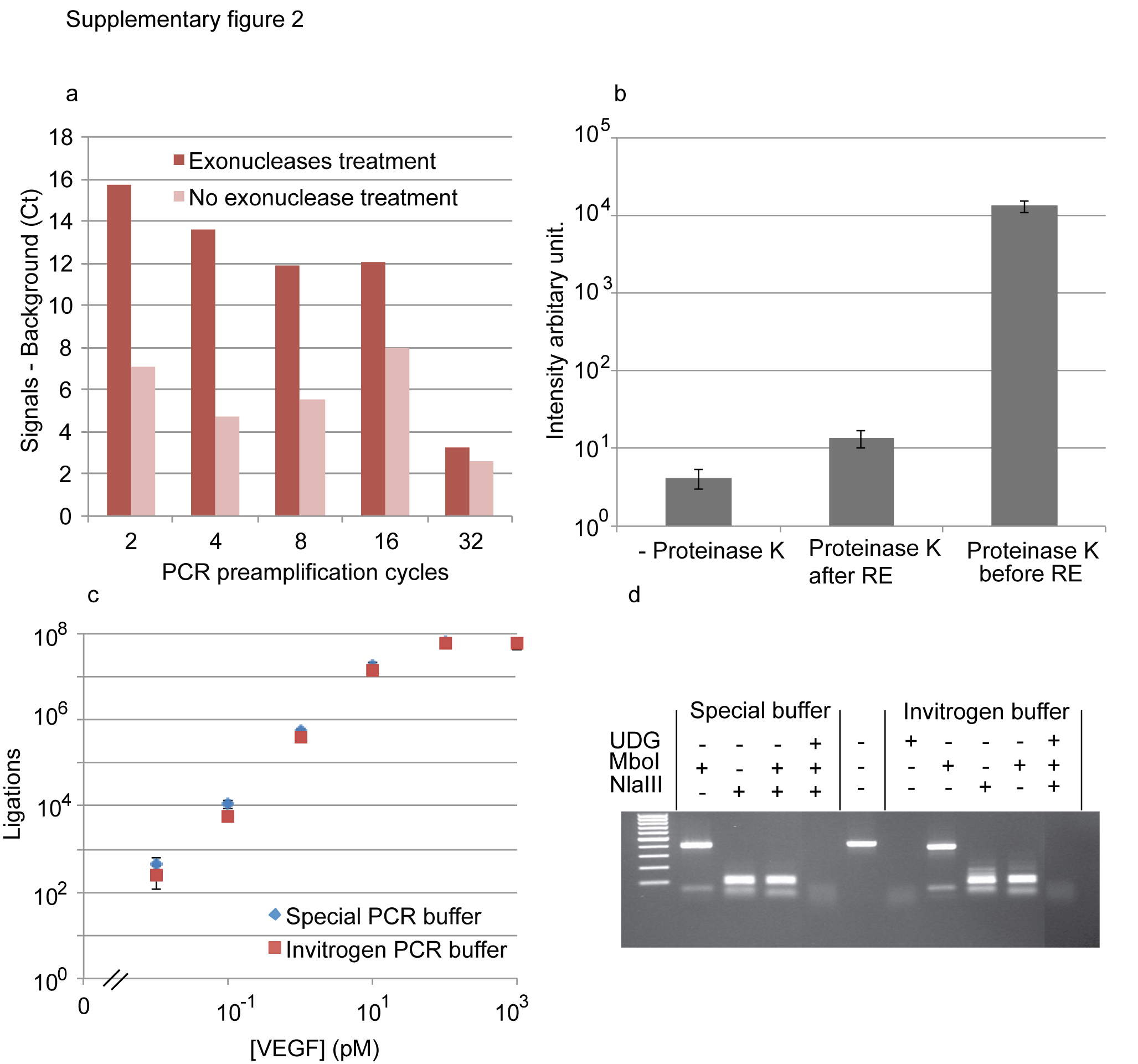

Supplement: Figure S2 — Optimization of the generation of reporter DNA strands. a) The assay background was significantly reduced by treating the ligation products with exonucleases and optimizing the number of PCR cycles. Two individual PLA reactions for detection of 10 pM VEGF were carried out with oligonucleotide system A1A2 and C1C2. After ligation, the ligation products were treated with a 50 µl exonuclease mix containing 0.1 U/µl lambda exonuclease and 0.2 U/µl phi29 polymerase, 0.01 µg/µl BSA in 1× special buffer (50 mM KAc, 3 mM MgAc, 20 mM Tris-HAc pH 7.5, 1 mM DTT) at 37°C for 30 minutes, followed by washing in 1× PBS, 0.05% Tween20. The treated products were pooled and amplified by PCR. Untreated ligation products were pooled and used as assay controls. The PCR-amplified products were diluted according to the results from analytical realtime-PCR quantification with specific primer pair combinations: AC and CC. Signals generated from primer combination AC are defined as background, while signals generated from primer combination CC are defined as signals. b) The effect of the Taq polymerase was investigated by addition of proteinase K at different points after PCR in comparison to reactions where no proteinase K was added. c) The buffer was optimized so that all enzymatic reactions could be performed in the same buffer. VEGF detection by PLA performing ligation and PCR amplification in the standard PCR buffer and the special buffer designed for the assay. d) The enzymatic efficiencies in the two separate buffers were investigated by treating PCR-products with the restriction enzymes NlaIII and MboI and UDG. (TIF) [file pone.0040405.s002.tif]

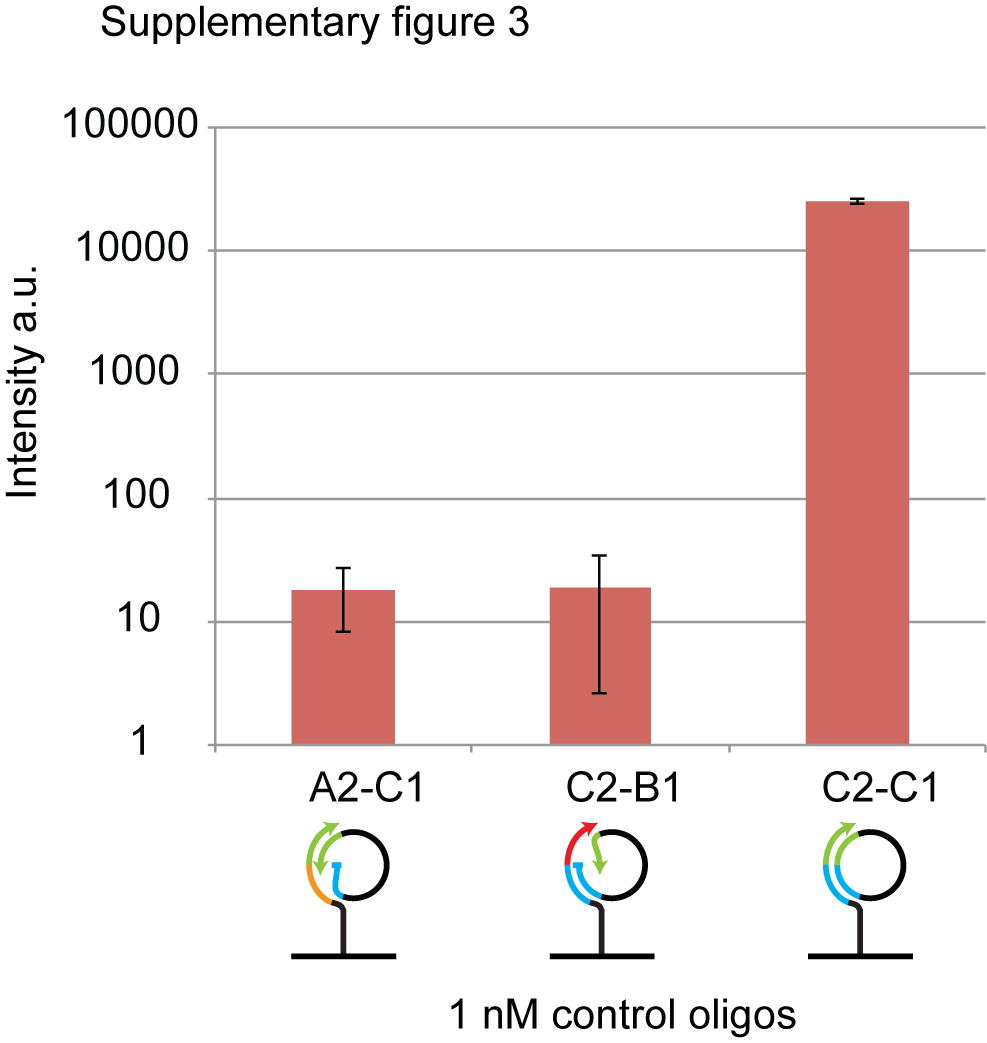

Supplement: Figure S3 — Comparison of signal from matched or partially mismatched reporter strands on DTM. The signal from the array spot with both tags complementary to the single stranded ligation product is significantly greater than for array features where only one of the tags is complementary. (TIF) [file pone.0040405.s003.tif]

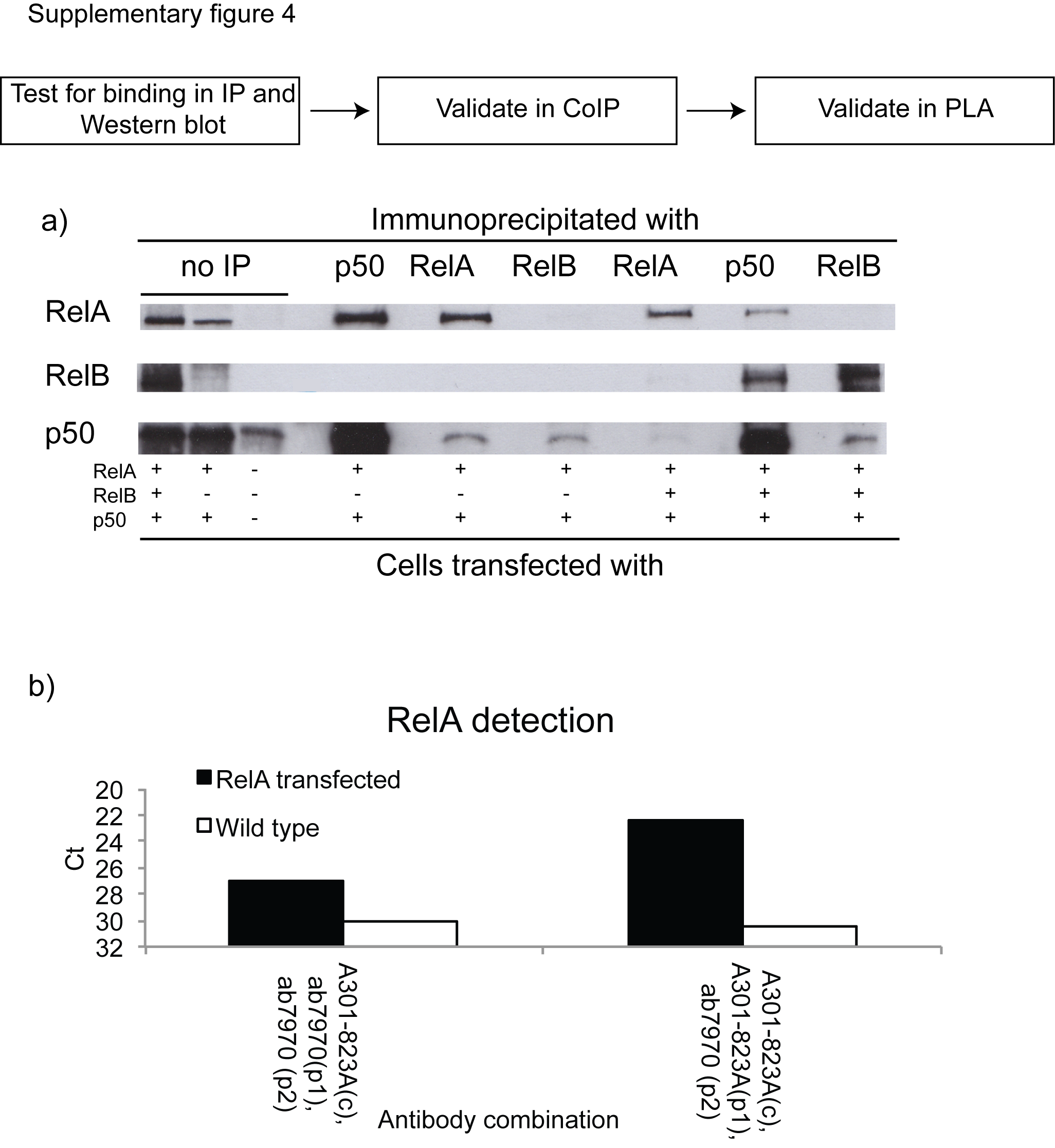

Supplement: Figure S4 — Antibody validation pipeline. All antibodies used for detection of NFκB-family proteins were validated by western blot to investigate if they could bind a protein of the estimated size, Co-IP to confirm that they could bind the native protein while interacting with its known interaction partners, and SP-PLA to find the best combination of binders to be used for PLA. a) Immunoprecipitation with antibodies against p50, RelA and RelB showed that the known interaction partners were co-immunoprecipitated together with the targeted protein. b) Different antibodies were used for capture (c), PLA probe 1 (p1) and probe 2 (p2) in SP-PLA. The combination with A301–823A as capture and one of the probes together with ab7970 as the other probe was superior in generating a high signal over background for detection of RelA when compared to using A301–823A as capture and ab7970 as the two PLA probes. (TIF) [file pone.0040405.s004.tif]

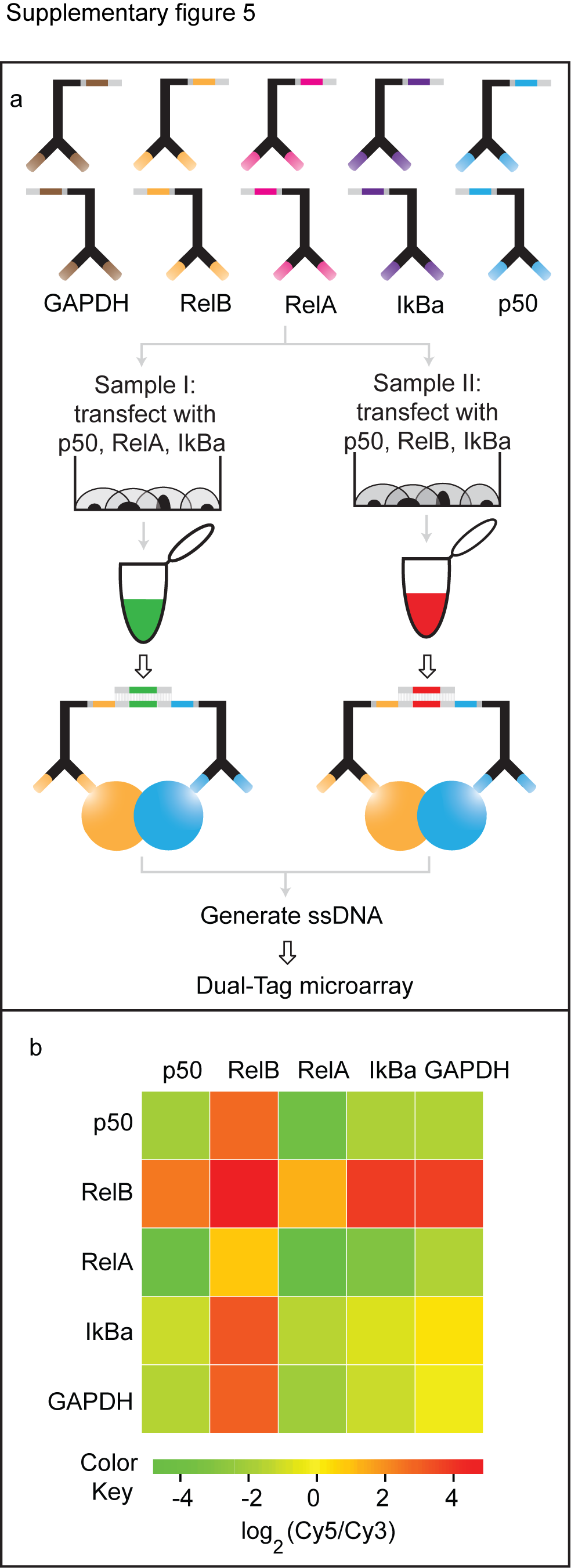

Supplement: Figure S5 — Validation of detection of protein-interaction between the NFκB-family proteins. The detection of protein interactions among the NFκB-family proteins was repeated with the two sample tag sequences, introduced during the ligation reaction, switched for the two samples. In this heat map negative log2 Cy5/Cy3 ratios (green) indicate a greater abundance in sample I (transfected with p50, RelA and IκBα), while positive log2 Cy5/Cy3 ratios (red) indicate a greater abundance in sample II (transfected with p50, RelB and IκBα). Raw data are presented in Table S1b. (TIF) [file pone.0040405.s005.tif]

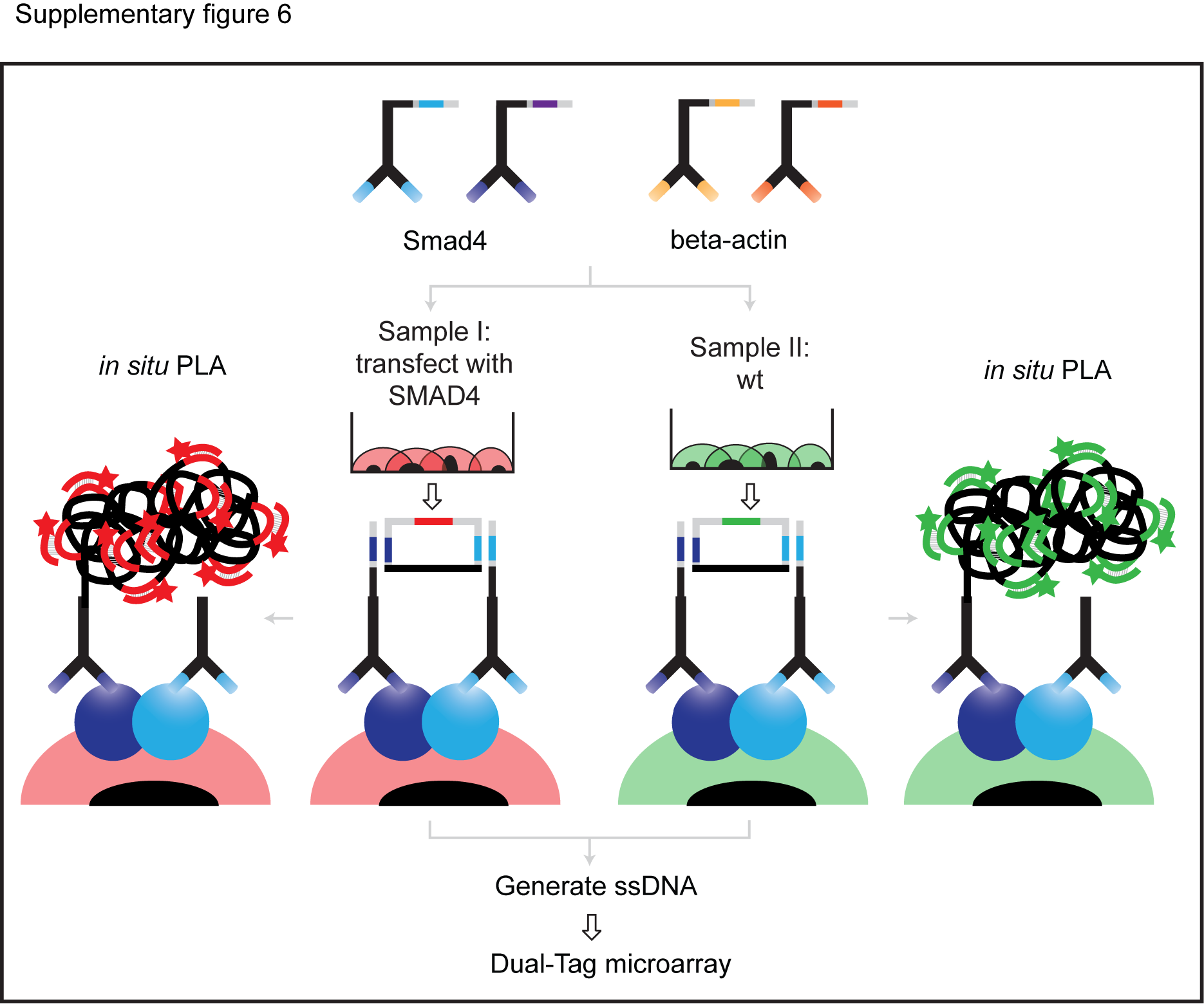

Supplement: Figure S6 — Experimental setup for in situ PLA detection of Smad4 with fluorescent in situ detection and DTM readout. PLA probes directed to Smad4 and beta-actin were applied to slides with fixed cells that had or had not been transfected with Smad4. Connector oligonucleotides were added and joined into circular reporter molecules by enzymatic ligation. The reporter DNA circles were detected in situ in cells fixed on slides by allowing them to guide rolling circle amplification to generate large concatemers of DNA. The amplification products were then detected by hybridization with a fluorescence labeled detection oligonucleotide. For an alternative readout on microarrays, reporter DNA circles were released from slides by treatment with proteinase K, amplified by PCR and then read out according to the protocol for DTM readout. (TIF) [file pone.0040405.s006.tif]

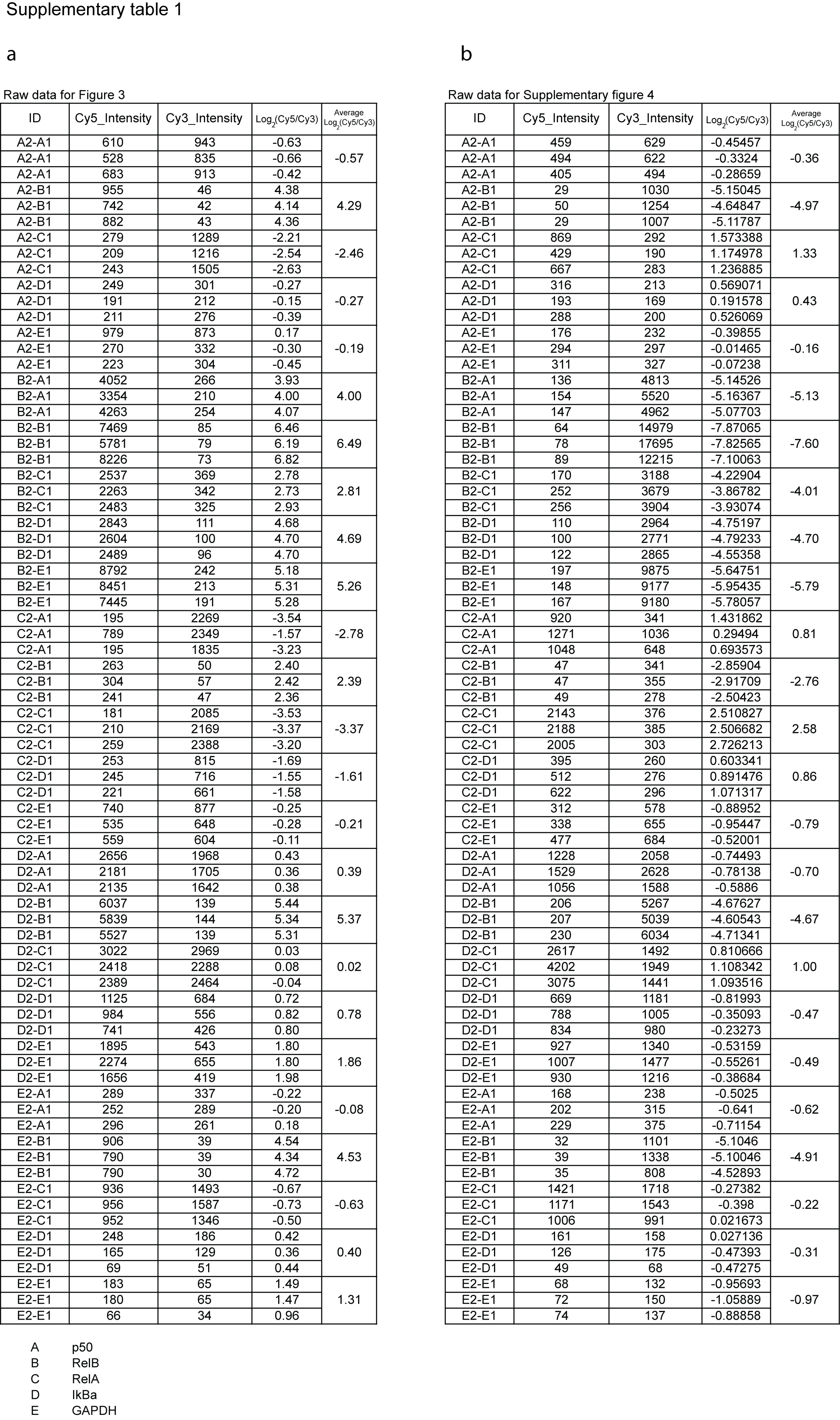

Supplement: Table S1 — Raw data for Figure 3 and Figure S5. Listed are the measured Cy5-intensities and Cy3-intensities for the experiments presented in a) Figure 3 and b) Figure S5. The ratios between the Log2(Cy5/Cy3) are calculated for individual array features, and an average value is calculated for the three replicate features within a subarray. (TIF) [file pone.0040405.s007.tif]

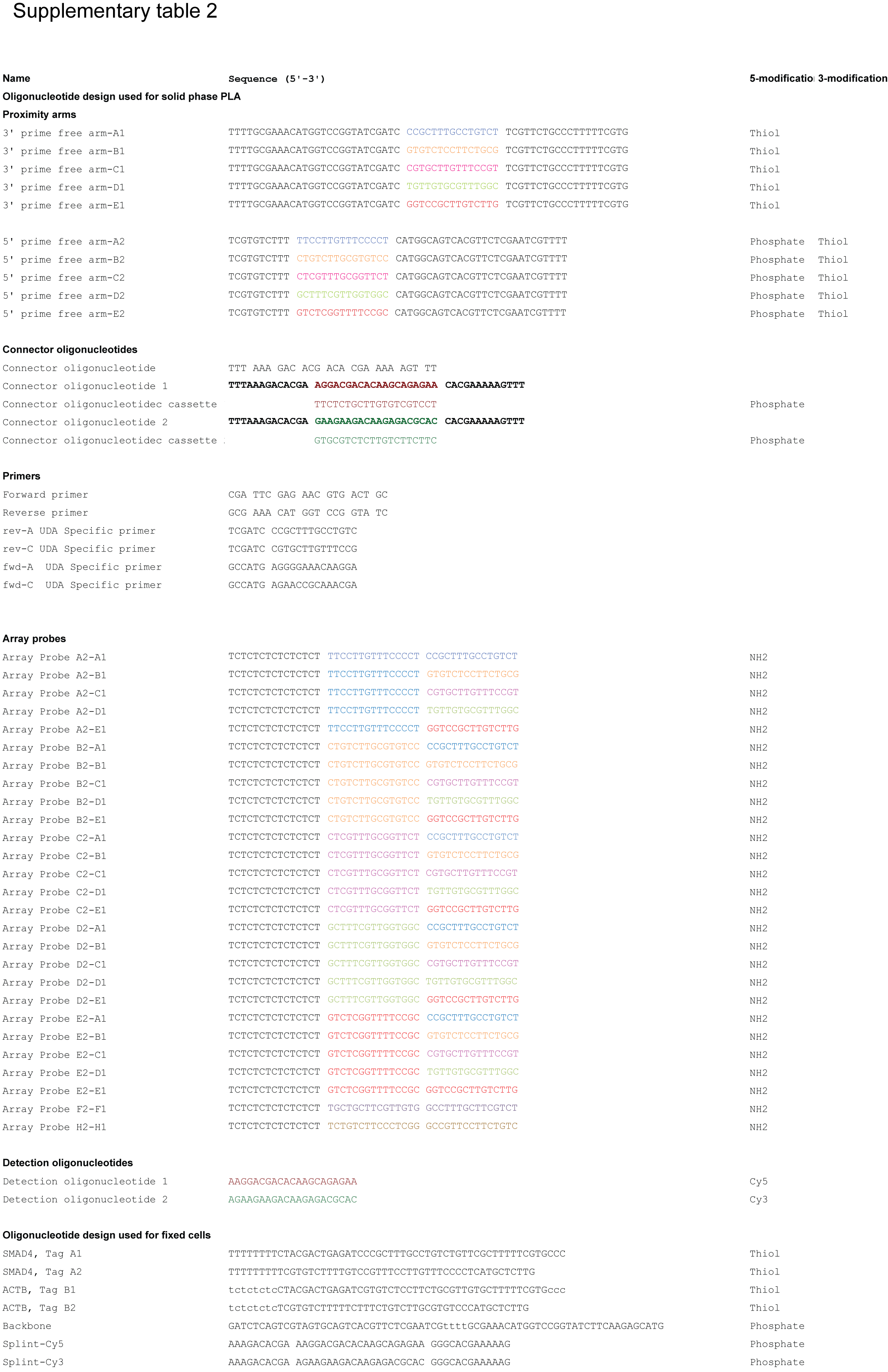

Supplement: Table S2 — List of oligonucleotides used in the experiments. Listed are the sequences of all oligonucleotides used in the experiments together with their modifications in the 3′-free and 5′-free ends respectively. (TIF) [file pone.0040405.s008.tif]

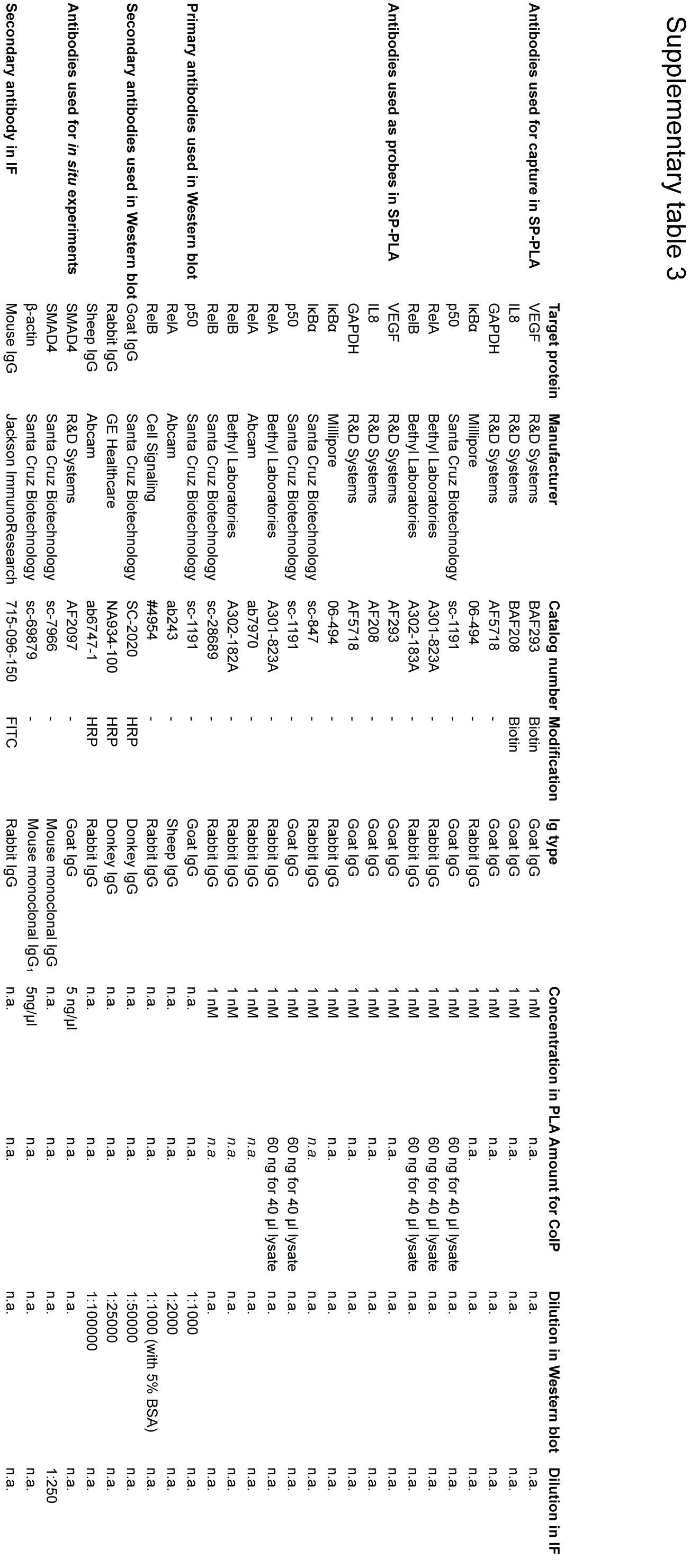

Supplement: Table S3 — List of antibodies used in the experiments. Listed are the antibodies with their target proteins, catalog number and manufacturer, any modification, type of immunoglobulin (Ig), and concentrations in various assays. (TIF) [file pone.0040405.s009.tif]
