# Supplementary material for: Profiling Cellular Protein Complexes by Proximity Ligation with Dual Tag Microarray Readout
Source: PLoS One. 2012 Jul 10;7(7):e40405. doi: 10.1371/journal.pone.0040405 (PMC3393744; doi:10.1371/journal.pone.0040405)
Supplement: File S1 — Supplementary Results and Materials and methods. (PDF) [file pone.0040405.s010.pdf]

Supplementary materials

RESULTS

Assay optimizations

The combination of PLA with DTM for multiplex detection of proteins and protein-protein interactions is illustrated in Figure S1. The assay is designed for direct comparison of two samples in the same spots on a DTM using a dual color readout. PLA is performed on immune-precipitates of molecules of interest from cell lysates, or on cells fixed on a microscope slide, using a variant of methods previously described [1,2] (Figure S1a, Figure S6). Reaction products from individual samples are labeled with one of two unique DNA sequences, allowing pairs of samples to be directly compared on microarrays via detection oligonucleotides labeled either with Cy3 or Cy5. This sample labeling is accomplished by interposing a short sample-specific sequence when pairs of PLA probes are joined by ligation (Figure S1b). All connector oligonucleotides used for templating ligation, and oligonucleotides on PLA probes that have not been incorporated into a ligation product are destroyed by exonuclease treatment (Figure S1c), to limit background signals (Figure S2a). Thereafter the ligated reporter molecules from the two samples to be compared are pooled into one reaction tube and amplified by PCR (Figure S1d). After digesting the Taq polymerase with proteinase K (Figure S2b) the unique tag sequences are displayed at the ends of the reporter molecules by cleaving the amplified reporter molecules at restriction sites included in all of the oligonucleotides attached to antibodies (Figure S1e). PCR amplification is performed substituting deoxy-uracil (dUTP) for deoxy-thymidine (dTTP). This serves to ensure that the reporter molecules (where one of the strands by design lacks T or U residues) are made single stranded prior to hybridization on the array by removing their complements by treatment with uracil-DNA glycosylase (UDG) (Figure S1f). The two end tags that are displayed due to the restriction enzyme treatment can then hybridize to the appropriate oligonucleotides on the arrays, allowing the reporter strands to be ligated into circles (Figure S1g). This is followed by localized amplification by RCA on the microarray (Figure S1h), as previously described [3]. We have optimized the buffer conditions so that PCR, restriction enzyme treatment, and UDG treatment can all be performed in the same buffer, eliminating the need to change buffers and thereby minimizing the risk of losing reporter molecules (Figure S2c-d).

The use of DTM has previously been shown to exhibit greater specificity of detection of target sequences compared to the more commonly used hybridization reactions on microarrays [3]. Here, the DTM concept was used to detect reporter molecules having two separate tag sequences. The requirement for ligation of the reporter molecules on the microarray ensured that only reporter molecules with both tags complementary to the oligonucleotides in a certain spot on the array could generate signals. We did not observe any cross-ligation between reporter molecules and array features where none or only one of the tags was complementary (Figure S3).

MATERIALS AND METHODS

Reagents

Plasmids

Full length cDNA of RelB (IOH11686) and full length cDNA of IκBα (IOH4138) each inserted into pdEYFP-C1amp vectors were purchased from imaGenes (Berlin, Germany). Full length cDNA of p50 (NFKB1) inserted into pcDNA3 vector was a kind gift from Dr. George Mosialos (Al. Fleming BSRC, Athens, Greece). Full length cDNA of RelA inserted into pDNR-Dual vector (clone ID HsCD00022126; source Harvard’s Institute of Proteomics) was obtained from the PlasmID repository (Harvard Medical School, Boston, MA). We inserted it into pLPS-AcGFP1-N with Cre recombinase according to the instruction for Creator™ DNA cloning kits (Clontech).

Antibodies and proteins

The antibodies used for PLA, western blot and immunofluorescence are listed in Table S3. The antibodies used for capture in SP-PLA, i.e. immobilized on the paramagnetic beads, had to be biotinylated. The antibodies for capture that was purchased without biotin were labeled with biotin using the Chromalink Biotin labeling kit (Solulink) according to manufacturer’s instructions.

Full length recombinant human VEGF (293-VE-050) and IL8 (208-IL-010) were purchased from R&D systems.

Preparation of PLA probes

PLA probes were prepared by conjugating the antibodies to oligonucleotides as previously described [2] with minor modifications. Briefly, the antibody at a concentration of 2 mg/ml in PBS was activated with a 30-fold molar excess of sulfosuccinimidyl-4-(*N*-maleimidomethyl) cyclohexane-1-carboxylate (sulfo-SMCC; Pierce) freshly dissolved in DMSO (Sigma-Aldrich) that was incubated for 2 h at room temperature (RT). Twenty mM oligonucleotides (Table S2) modified with thiol groups at the 3' or 5' ends were reduced with 80 mM DTT (Sigma-Aldrich) in 0.8X PBS with 4 mM EDTA for 1 hour at 37°C. We purified the antibody from free sulfo-SMCC and the oligonucleotides from DTT using three consecutive MicroSpin G-50 columns each (GE Healthcare) that had been equilibrated with 5 mM EDTA in 1X PBS. The oligonucleotide was mixed with the antibody at a 2.5-fold molar excess. The reaction was allowed to proceed over night at 4°C during dialysis (Slide-A-Lyzer Mini dialysis units 7K MWCO, Pierce) against 1X PBS.

Quantitative PCR readout of SP-PLA

For quantitative readout or the SP-PLA, the ligation was performed in the same mix as the PCR. Thus, after washing away unbound PLA probes a ligation and PCR mix containing 50 mM KAc, 20 mM Tris-HAc pH 7.5, 3 mM MgAc, 1 mM DTT, 0.2 mM dNTPs containing dUTP instead of dTTP (Fermentas), 0.1 U UDG (Fermentas), 1.5 U platinum Taq polymerase (Invitrogen) and 100 nM of forward and reverse universal PCR primers, and connector oligonucleotide (Table S2), 0.5 X SYBR Gold (Molecular Probes), 0.08 mM ATP (Fermentas), and 0.01 U/µl T4 DNA ligase was added to the beads with captured antigen and PLA probes. Amplification was carried out by incubating at 95°C for 2 min, followed by 40 cycles of 95°C for 15 sec and 60°C for 1 min. Quantitative PCR was performed using a Stratagene MX 3000P instrument.

Production of oligonucleotide microarrays

Microarrays were produced using a Piezorray system (PerkinElmer Life and Analytical Sciences, Inc. MA, USA). Each microarray comprised 16 subarrays printed with 9 mm spacing in both the x- and y-axis. Oligonucleotides (Table S2) were printed in triplicates in each subarray. All microarrays were printed on Codelink slides (GE Healthcare Europe, GmbH, Germany), followed by incubation in a humidity chamber. The slides were blocked

for 5 min in 100 ml PBS, 20% ethanol, 0.05 M $NaBH_4$ at RT, rinsed in water, and dried by centrifugation.

Individual subarrays were separated from each other during experiments using a silicone rubber mask to address individual subarrays for enzymatic and hybridization reactions as previously described [4] with minor modifications. Briefly, an inverted 96-well microtiter plate with V-shaped wells was used as a mold and liquid silicon rubber (Elastosil RT 601 A/B, Wacker-Chemie GmbH, Munich, Germany) was poured into the mold, leaving the tips of the wells uncovered. When the rubber had hardened, the big silicon mask containing 96 cone-shaped reaction chambers was cut to match the size of microscope slides with 16 wells per mask, one reaction chamber for each subarray. To enable fixation of the silicon mask to the glass slide, the silicon mask was washed with 1% Alconox (Alconox) and then firmly pressed against the glass surface and put between two custom-made acrylic glass plates, the upper one with drilled holes for the pipette tips. During incubations the holes in the upper acrylic glass plate were covered with sealing tape (Sarstedt) to avoid evaporation.

Cell culture, transfection and lysis

Human embryonic kidney 293T cells [5] were obtained and cultured according to protocols from the American Type Culture Collection (ATCC). Cells were transiently transfected with the NFκB constructs by calcium phosphate transfection. For one 35 mm plate with approximately 50% confluent cells, 1 µg plasmid DNA for each protein to be transfected was diluted to a total volume of 90 µl with sterile water. One hundred µl 2X BES buffered saline solution (50 mM N,N-bis(2-hydroxyethyl)-2-aminoethanesulfonic acid, 280 mM NaCl, 1.5 mM Na_2HPO_4; pH 6.9) was added followed by addition of 10 µl 2.5 M $CaCl_2$. The mixture was vortexed and left standing in RT for 30 min before added drop wise to the cells. After incubation of the cells for 4 hours at 37°C the medium was changed. The transfected cells were allowed to grow for 24 hours and were then lysed. The cells were first rinsed with 1X PBS and then lysed in lysis buffer (10 mM Tris pH 7.4, 150 mM NaCl, 0.5% NP40, 1% TritonX-100, 1 mM EDTA, 1 mM EGTA) supplemented with cOmplete Mini Protease Inhibitor Cocktail, and PhosSTOP Protease Inhibitor Cocktail (1 tablet of each per 10 ml lysis buffer; Roche Applied Science). The protein concentration was determined with the BCA Protein Assay kit (Pierce) according to manufacturer’s instructions. Prior to any PLA the cell lysate samples were diluted to have the same total protein concentration.

Co-immunoprecipitation and western blot

Eight µl of the beads used for capture in SP-PLA (i.e. 40 µg beads with 60 ng antibody) was used for immunoprecipitation of 40 µl of cell lysate. The beads were blocked in PLA buffer for 20 min in RT with end-over-end rotation prior to mixing with the cell lysate. The precipitation was proceeded over night at 4°C. The beads were washed four times with 100 µl cell lysis buffer. The precipitated proteins were eluted in 30 µl Laemmli buffer (Laemmli sample buffer (Bio-Rad) with 5% freshly added β-mercaptoethanol (Sigma)) during incubation at 95°C for 5 min.

The Criterion pre-cast gel system (Bio-Rad) was used for electrophoresis and blotting according to manufacturer's instructions. Cell lysates that were not precipitated were mixed 1:1 with Laemmli buffer and incubated at 95°C for 5 min. Thirty µl of each sample was loaded on a 7.5% Tris-glycine gel and the gel was run at 170V. The proteins were transferred to a Hybond-C Extra RPN303E membrane (GE Healthcare). After blotting the membrane was blocked with 10% powder milk (Semper) in TBS-T (1X TBS with 0.1% Tween-20) at 4°C over night. The primary antibodies were incubated with the membrane one at the time diluted in TBS-T according to Table S3, at 4°C over night. The membrane was washed five times in TBS-T prior to applying the horseradish peroxidase (HRP) conjugated secondary antibodies (diluted in TBS-T according to Table S3) and incubated for 1 h at RT. The membranes were washed five times in TBS-T and were then developed by enhanced chemiluminescence (ECL), prepared by mixing 15 µl 0.1% Luminol and 0.02% p-Coumaric acid in 0.1M Tris-HCl pH 8.5 with 5 ml 0.02% H_2O_2 in 0.1 M Tris-HCL pH 8.5 (Sigma-Aldrich Sweden AB) immediately before the development.

In situ *visualization of PLA products by RCA*

For *in situ* imaging of the PLA assay, DNA circles formed by ligation were amplified in 0.125 U/µl phi29 DNA polymerase (Fermentas), 50 mM Tris-HCl (pH 7.5), 10 mM $MgCl_2$, 10 mM $(NH_4)_2SO_4$, 250 mM dNTPs, 0.25 µg/µl BSA, 0.05% Tween-20 and 5% glycerol, and incubating at 37°C for 90 min, followed by two 2 min washes in TBS with 0.05% Tween-20 at RT. RCA products were detected by adding detection oligonucleotide 1 (Table S2) at 6.25 nM, in a mixture comprising 2X SSC, 0.25 µg/µl BSA, 7.5 ng/µl poly (A) and 0.05% Tween-20. Smad4 was detected by immunofluorescence by staining with a mouse monoclonal

antibody (Table S3) diluted 1:250, followed by incubation with FITC-labeled rabbit-anti-mouse antibody, and nuclei were visualized by staining with 1mM Hoechst 33342 (Sigma). After two 5 min washes in TBS with 0.05% Tween-20 and one 5 min wash in 0.2X SSC, slides were mounted on a cover slip with Vectashield (CliniScience, Montrouge) and sealed with nail polish. Images were acquired by an epifluorescence microscope (Axioplan II, Zeiss) and processed by the AxioVision LE 4.3 software (Zeiss).

References

1. Darmanis S, Nong RY, Hammond M, Gu J, Alderborn A, et al. (2010) Sensitive plasma protein analysis by microparticle-based proximity ligation assays. Mol Cell Proteomics 9: 327–335. doi:10.1074/mcp.M900248-MCP200.

2. Soderberg O, Gullberg M, Jarvius M, Ridderstrale K, Leuchowius K-J, et al. (2006) Direct observation of individual endogenous protein complexes in situ by proximity ligation. Nat Meth 3: 995–1000. doi:10.1038/nmeth947.

3. Ericsson O, Jarvius J, Schallmeiner E, Howell M, Nong RY, et al. (2008) A dual-tag microarray platform for high-performance nucleic acid and protein analyses. Nucl Acids Res 36: e45. doi:10.1093/nar/gkn106.

4. Pastinen T, Raitio M, Lindroos K, Tainola P, Peltonen L, et al. (2000) A System for Specific, High-throughput Genotyping by Allele-specific Primer Extension on Microarrays. Genome Research 10: 1031–1042. doi:10.1101/gr.10.7.1031.

5. DuBridge RB, Tang P, Hsia HC, Leong PM, Miller JH, et al. (1987) Analysis of mutation in human cells by using an Epstein-Barr virus shuttle system. Mol Cell Biol 7: 379–387.
