# Supplementary figures and images for: Correction: Profiling Cellular Protein Complexes by Proximity Ligation with Dual Tag Microarray Readout
Source: PLoS One. 2015 Mar 25;10(3):e0119890. doi: 10.1371/journal.pone.0119890 (PMC4373876; doi:10.1371/journal.pone.0119890)

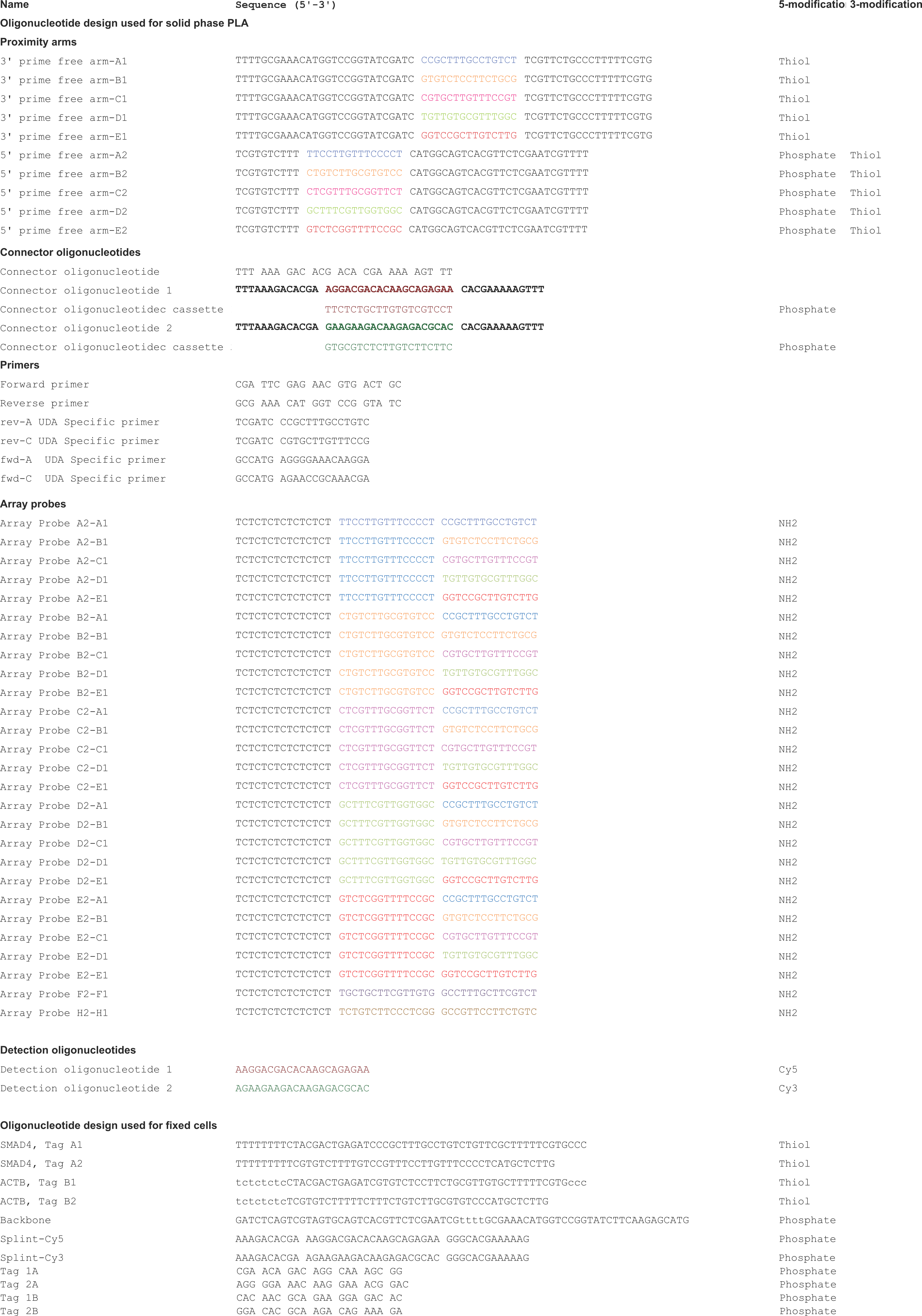

Supplement: S2 Table — Listed are the sequences of all oligonucleotides used in the experiments together with their modifications in the 3′-free and 5′-free ends respectively. (TIF) [file pone.0119890.s001.tif]
